# Mediterranean Diet and Neurodegenerative Diseases: Recent Advances from the Gut–Immune–Brain Axis to Multi-Omics-Guided Precision Nutrition

**DOI:** 10.3390/cells15141287

**Published:** 2026-07-17

**Authors:** Jiaxing Dou, Jiahui Wang, Shihan Chen, Elizabeth B. Hsueh, Isabella L. Scott, Feng Xue

**Affiliations:** 1 Pathology and Pharmacology Laboratory for Innovative Research, Hwamei College of Life and Health Sciences, Zhejiang Wanli University, Ningbo 315100, China; 17614756601@163.com (J.D.); wangjiahui202507@163.com (J.W.); 18281195468@163.com (S.C.); 2 Department of Biology, Texas Biopharma Instrument LLC, Frisco, TX 75035, USA; elizabeth.hsueh@txbpi.cc (E.B.H.); isabella.scott@txbpi.cc (I.L.S.); 3 Zhejiang Key Laboratory of Intelligent Food Logistic and Processing, Yuyao Innovation Institute, Zhejiang Wanli University, Ningbo 315400, China

**Keywords:** neurodegenerative diseases, mediterranean diet, neuroinflammation, gut–immune–brain axis, gut microbiota, precision nutrition, multi-omics, systems biology

## Abstract

Neurodegenerative diseases like Alzheimer’s disease (AD) and Parkinson’s disease (PD) are regarded as systemic illnesses, mainly characterized by long-term neuroinflammation, metabolic dysregulation and barrier dysfunction. All these factors interact through the gut–immune–brain axis. The Mediterranean diet, rich in plant-based foods, olive oil, and fish, has been consistently associated with slower cognitive decline and reduced risk of neurodegeneration in observational studies and some clinical trials. This review provides a systems-level synthesis that distinguishes itself from previous narratives by integrating disease-specific mechanistic frameworks with multi-omics-guided precision nutrition strategies. We summarize recent evidence showing that this dietary pattern can remodel gut microbiota composition and enhance the production of bioactive metabolites such as short-chain fatty acids (SCFAs). These metabolites are associated with improved intestinal barrier integrity, reduced systemic inflammation, and potential modulation of brain functions. Within the central nervous system, diet-related metabolites have been linked to reduced neuroinflammation via modulation of microglial and astrocytic states. They have been linked to protection of mitochondrial function, maintenance of proteostasis, and preservation of blood–brain barrier (BBB) integrity. In AD and PD patients, adherence to this diet is associated with favorable changes in pathological hallmarks, including amyloid-beta (Aβ), tau, and α-synuclein accumulation. Nowadays, multi-omics tools, including single-cell transcriptomics, spatial transcriptomics and microbiome analysis, are widely used in this field, which helps researchers explore these complicated effects more deeply. Importantly, individual responses to the diet vary considerably due to differences in genetic background, gut microbial composition, and metabolic phenotypes, which underscore the need to move from generalized dietary guidelines toward personalized precision nutrition. The Mediterranean diet is not only a dietary pattern but also an effective way to modulate neuro-immune and metabolic networks. However, current evidence remains largely observational, and we critically discuss the need for more randomized controlled trials (RCTs) and standardized multi-omics data analysis frameworks. To sum up, the Mediterranean diet plays a neuroprotective role via the gut–immune–brain axis, and multi-omics techniques promote the development of precision nutrition. More trials and improved multi-omics systems are required to apply these research results in clinical practice.

## 1. Introduction

Neurodegenerative diseases, such as Alzheimer’s disease (AD) and Parkinson’s disease (PD), are characterized by complex, multifactorial pathogenesis and are closely associated with chronic inflammation and oxidative stress [1]. These disorders now represent a major public health challenge worldwide. A 2025 report from the Alzheimer’s Association provides striking epidemiological data: in the United States, approximately 7.2 million individuals aged 65 and older are living with AD, a figure projected to reach 13.8 million by 2060. Notably, between 2000 and 2022, the age-adjusted death rate from AD increased by more than 142%, while death rates from heart disease, stroke, and AIDS declined during the same period [2].

Notably, neurodegenerative diseases represented by AD are not determined solely by genetic factors but rather arise from the interplay between genetic predisposition and multiple modifiable factors. Epidemiological studies have demonstrated that nutrition, environmental exposures, and lifestyle can significantly influence the onset, progression, and treatment response of these disorders, with modifiable risk factors estimated to account for approximately 45% of worldwide dementias. These modifiable factors affect central nervous system homeostasis through shared pathways, including oxidative stress, neuroinflammation, mitochondrial dysfunction, and dysregulation of the gut—brain axis, thereby providing a theoretical basis for multitarget dietary interventions [3,4]. The Mediterranean diet (MedDiet) is a plant-based dietary pattern characterized by high consumption of fruit, vegetables, olive oil, legumes, nuts and fish; moderate consumption of red wine; and low consumption of red meat, processed foods and sugar-sweetened products [5]. Among modifiable dietary patterns, the Mediterranean diet has emerged as particularly relevant for brain health in middle-aged and older adults. Two other dietary patterns—the Dietary Approaches to Stop Hypertension (DASH) diet and the Mediterranean-DASH Intervention for Neurodegenerative Delay (MIND) diet—have also been associated with cognitive benefits. However, the Mediterranean diet demonstrates the most consistently observed protective relationship with brain health across diverse populations. In contrast, the Western dietary pattern is characterized by high consumption of ultra-processed foods, refined carbohydrates, added sugars, saturated and trans fats, and low intake of dietary fiber and polyphenols. This dietary profile promotes systemic inflammation through multiple pathways, including endotoxemia from gut barrier disruption, activation of pro-inflammatory transcription factors such as NF-κB, and induction of oxidative stress. Consequently, the Western diet is associated with an elevated risk of cognitive decline and neurodegeneration. Key components of the Western diet—saturated fats, refined sugars, and processed meats—promote systemic inflammation and metabolic dysfunction through distinct mechanisms. Processed meats supply precursors for microbial Trimethylamine N-oxide (TMAO) production, which exacerbates Aβ aggregation and BBB dysfunction. Meanwhile, the low fiber content of the Western diet reduces SCFA production, compromising gut barrier integrity and facilitating systemic inflammation. Collectively, these component-specific effects illustrate how the Western dietary pattern may actively contribute to neurodegenerative pathogenesis. The mechanistic contrast between the Mediterranean and Western dietary patterns underscores the importance of dietary quality in modulating neuroinflammatory and neurodegenerative processes. Stroke, dementia, and related disorders share common pathophysiological mechanisms, including inflammation, oxidative stress, and vascular damage. Given its multitarget actions, the Mediterranean diet may exert neuroprotective relationship through these convergent pathways, providing a nutritional foundation for disease prevention [6,7,8].

To contextualize the subsequent mechanistic discussions, it is important to define what constitutes the Mediterranean diet. Although not a single rigid prescription, the Mediterranean diet is traditionally derived from the culinary practices of countries bordering the Mediterranean Sea. It is consistently characterized by high consumption of plant-based foods including vegetables, fruits, whole grains, legumes, nuts, and seeds, liberal use of extra virgin olive oil as the principal fat source, moderate intake of fish, poultry, and dairy products, low consumption of red and processed meats and sweets, and moderate wine consumption typically with meals [1]. From a nutritional biochemistry perspective, key bioactive constituents include monounsaturated fatty acids, n-3 polyunsaturated fatty acids, dietary fiber, and diverse polyphenolic compounds. The neuroprotective relationship attributed to the Mediterranean diet are thought to arise from synergistic interactions among these multiple bioactive constituents rather than from any single component [9].

The Mediterranean diet has well-known direct neuroprotective properties, including polyphenols and unsaturated fatty acids. However, this review focuses on recent breakthroughs about the gut microbiota as a key mediator in the gut–immune–brain axis. This provides a new way to understand how the diet protects the brain systemically.

So the Mediterranean diet should be seen not solely as a nutritional pattern but as a modifiable intervention that works at the systems level. This review aims to bring together recent epidemiological and mechanistic evidence. We look at how the diet affects neurodegenerative diseases, summarize its bioactive components and protective mechanisms, and suggest future research directions.

As this review aims to provide an integrated systems-level synthesis rather than a quantitative meta-analysis, we conducted a narrative review of the literature. To ensure comprehensive coverage of relevant evidence, we searched PubMed and Web of Science for peer-reviewed articles published up to December 2025, using combinations of the following keywords: ‘Mediterranean diet,’ ‘neurodegenerative diseases,’ ‘Alzheimer’s disease,’ ‘Parkinson’s disease,’ ‘gut microbiota,’ ‘gut–brain axis,’ ‘neuroinflammation,’ ‘multi-omics,’ and ‘precision nutrition.’ We prioritized high-quality evidence including large-scale prospective cohort studies, randomized controlled trials, systematic reviews, and meta-analyses, while also incorporating mechanistic insights from preclinical studies where human data were limited. Given the broad and rapidly evolving nature of this topic, we selected studies that provided the most robust and mechanistically informative contributions to the gut–immune–brain axis framework, with an emphasis on recent publications (2018–2026). Reference lists of retrieved articles were also manually screened to identify additional relevant studies.

Throughout this review, we categorize mechanistic evidence into three tiers to help readers interpret the strength of the underlying evidence: (1) established mechanisms supported by human interventional or prospective data; (2) plausible pathways derived from preclinical models (e.g., animal or cell studies) that await human validation; and (3) speculative hypotheses generated from computational or in vitro studies requiring experimental confirmation. We explicitly indicate the evidence tier when discussing each proposed mechanism.

## 2. Mediterranean Diet: A Systems-Level Regulation Approach Through the Gut–Immune–Brain Axis

### 2.1. Gut Microbiota Remodeling and Functional Reprogramming

Dietary fiber, polyphenols, and omega-3 fatty acids (n-3 PUFAs) in the Mediterranean diet can optimize the structure of the gut microbiota [7]. These compositional changes represent the first tier of a hierarchical cascade—what we term the “Microbiome-Metabolite Axis”—which constitutes the primary mechanism through which dietary patterns interface with host physiology. A recent systematic review reported that the Mediterranean diet increases beneficial bacteria like *Faecalibacterium prausnitzii* and *Bifidobacterium*. At the same time, it reduces pro-inflammatory taxa. A 6-year cohort study provided further evidence. People who followed the Mediterranean diet closely had more short-chain fatty acid (SCFA)-producing bacteria and fewer pro-inflammatory microorganisms. Both high diet adherence and these microbial changes were linked to slower cognitive decline [10].

Butyrate is not only a microbial metabolite. It also works as a histone deacetylase inhibitor. This means it can change gene expression in the gut, in immune tissues linked to the gut, and even in the nervous system. Studies in animal models show that sodium butyrate treatment can stop neuronal cell death in PD models. In models of AD and traumatic brain injury, butyrate has also been linked to better learning and memory [11]. The gut microbiota changes caused by the Mediterranean diet are not random. They represent a shift in the microbial metabolic network. The network moves from a pro-inflammatory profile to one that produces SCFAs. This kind of functional change matters more for biology than just changes in the abundance of single bacterial types.

Some bacteria, like *Bacteroides*, have anti-inflammatory properties. They are known to protect against cognitive impairment. People with AD tend to have less *Bacteroides* in their gut microbiota. The fiber and polyphenols in the Mediterranean diet can help *Bacteroides* grow [12]. Together, these changes in gut bacteria provide the microbial basis for how the gut–brain axis works.

### 2.2. Microbiota-Derived Metabolites: The Functional Output of the Microbiome-Metabolite Axis

By modulating both the composition and function of the gut microbiota, the Mediterranean diet induces the production of distinct secondary metabolites, which in turn regulate three major metabolic networks.

#### 2.2.1. SCFAs

SCFAs are made mostly of butyrate, propionate, and acetate. They help the intestinal barrier work better. They also lower systemic inflammation and protect the brain through the vagus nerve-mediated gut–brain axis [6]. SCFAs do not only work in the gut. They can also cross the blood–brain barrier (BBB), especially butyrate. Once in the brain, they take part in epigenetic regulation by blocking histone deacetylase activity. This promotes synaptic plasticity and neurogenesis [13]. In some cases, like in germ-free conditions, SCFAs may actually promote Aβ deposition [11]. This evidence is derived primarily from animal models and human observational studies (Tier II); the direct causal role of SCFAs in Mediterranean diet-mediated neuroprotection in humans has not yet been established through RCTs.

#### 2.2.2. Secondary Bile Acids and TMAO

TMAO makes AD pathology worse by helping Aβ aggregate and by disrupting the blood–brain barrier (BBB). Clinical studies have found that people with AD have lower levels of SCFAs and indole-related metabolites, and higher levels of TMAO. These changes are linked to scores on cognitive tests [8]. TMAO is a gut microbiota-derived metabolite generated from dietary choline and L-carnitine, which are abundant in red meat and eggs. Gut microbes metabolize these precursors to trimethylamine, which is subsequently oxidized to TMAO in the liver. Preclinical studies have shown that TMAO promotes Aβ aggregation and disrupts blood–brain barrier integrity, and elevated TMAO levels have been observed in Alzheimer’s disease patients in association with cognitive decline. Dietary patterns significantly influence TMAO production: the Mediterranean diet is associated with reduced TMAO generation, whereas Western-style diets promote higher TMAO levels. Secondary bile acids, including deoxycholic acid, lithocholic acid, and ursodeoxycholic acid, are generated through microbial biotransformation of primary bile acids. These metabolites activate the farnesoid X receptor and TGR5 signaling pathways, which regulate metabolic and immune responses to reduce neuroinflammation, and their composition is influenced by dietary patterns, with Mediterranean diet components—particularly dietary fiber and polyphenols—promoting a favorable profile [6]. The roles of TMAO and secondary bile acids in AD pathogenesis are supported by preclinical and human correlational studies (Tier II); direct evidence linking Mediterranean diet-induced changes in these metabolites to clinical outcomes is currently lacking.

#### 2.2.3. Tryptophan Metabolites

Tryptophan metabolism goes through three main pathways: kynurenine, serotonin, and the microbiota-derived indole pathway. Metabolites from the indole pathway, like Indole-3-propionic acid (IPA) and indole-3-acetic acid, help control gut barrier integrity, immune signaling, and host–microbiota interactions. IPA protects the brain by activating the aryl hydrocarbon receptor (AhR) pathway. The Mediterranean diet is rich in fiber and polyphenols. It may boost beneficial indole metabolites and, through anti-inflammatory effects, reduce excessive kynurenine pathway activity. This increases tryptophan availability and serotonin synthesis. The diet, especially with extra virgin olive oil (EVOO), contains polyphenols that may directly raise plasma tryptophan levels. Anti-inflammatory effects from gut metabolites like SCFAs may also indirectly affect the kynurenine pathway [8,14]. Epidemiological evidence from a case–cohort study (*n* = 805) demonstrated that seven gut microbiome-derived metabolites, including tryptophan indolic metabolites (5-hydroxyindole-3-acetic acid, indole-3-butyric acid, indole-3-acryloylglycine, indole-3-lactic acid, indole-3-acetic acid methyl ester) and branched-chain SCFAs (isobutyric acid, 2-methylbutyric acid), discriminated incident dementia cases from non-cases, with 5-hydroxyindole-3-acetic acid significantly associated with time-to-dementia [15]. The tryptophan-indole-AhR pathway has been demonstrated in animal models and is supported by human AD hippocampal data (Tier II); however, whether Mediterranean diet directly modulates this pathway in patients remains to be tested.

### 2.3. Intestinal Barrier Integrity and Systemic Inflammatory Response

The second core axis—the Gut Barrier-Systemic Inflammation Axis—links microbial metabolites to peripheral immune status. The Mediterranean diet may also protect the brain by working on the gut–brain axis. It has high levels of fiber, polyphenols, and n-3 PUFAs. These can improve gut microbial composition and enhance SCFA production (detailed in Section 2.2.1), thereby strengthening the intestinal barrier and the blood–brain barrier (BBB), and lowering systemic inflammation caused by lipopolysaccharide. As a result, this reduces neuroinflammation, helps clear Aβ, and affects neurotransmitter synthesis. Through these multiple pathways, the diet may slow down neurodegenerative progression [10]. In addition, phytosterols may indirectly reduce the risk of vascular dementia by lowering cholesterol levels [12]. This lowering of peripheral inflammation also helps reduce neuroinflammation in the brain. This axis is particularly relevant because peripheral inflammation serves as a critical bridge connecting gut-derived signals to central nervous system pathology.

**Figure 1:**
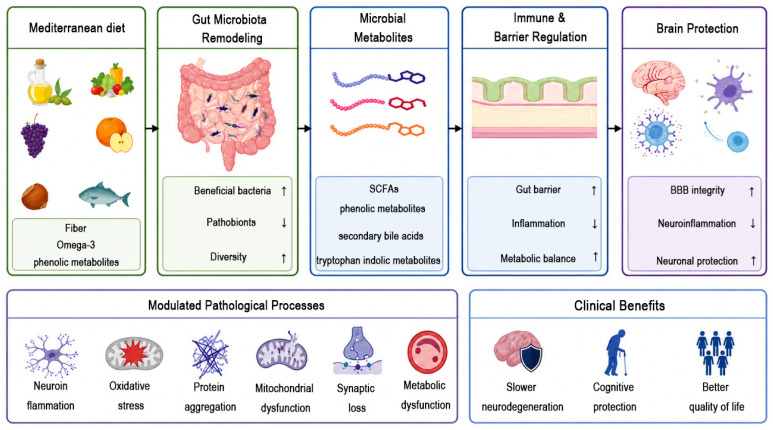
Mediterranean diet-mediated gut–immune–brain axis remodeling in neurodegenerative disorders. Schematic illustration of the proposed mechanisms by which Mediterranean diet-associated nutritional components modulate neurodegenerative disease progression through the gut–immune–brain axis. Dietary bioactive compounds reshape gut microbiota composition by increasing beneficial microbial populations and suppressing pathobionts, thereby enhancing microbial diversity and metabolic homeostasis. These alterations promote the production of neuroprotective microbial metabolites, including SCFAs, polyphenol-derived metabolites, bile acid intermediates, and tryptophan metabolites. Subsequently, improved intestinal barrier integrity and immune regulation reduce systemic inflammation and metabolic dysregulation, leading to decreased neuroinflammatory signaling and enhanced brain protection. Downstream pathological processes influenced by these mechanisms include oxidative stress, protein aggregation, mitochondrial dysfunction, synaptic degeneration, and vascular/metabolic impairment. Collectively, these coordinated effects contribute to cognitive protection, delayed neurodegeneration, and improved quality of life.

Recent studies show that microglia link gut bacteria changes to cognitive function. Human and transplant studies find that exosomes from obesity-related gut bacteria briefly activate microglia after one hour. But after 24 h, microglia become exhausted, with lower Triggering receptor expressed on myeloid cells 2 (TREM2), more inflammatory cytokines (TNF-α, IL-6, IL-1β), and poor phagocytosis. In contrast, transplant after alternate-day fasting gives microglia a healthy shape, better phagocytosis, more cell renewal (Ki67^+^), less p16 and PFKFB3, lower IL-6 and IL-1β, and better memory. This puts microglia at the center of the gut–immune–brain axis and shows how diets like the Mediterranean diet protect the brain [16] (Figure 1).

Beyond the microbiota-mediated effects above, polyphenols and other bioactive components in the Mediterranean diet may also act directly on the central nervous system. Lab and animal studies show that polyphenols may help neural stem cells grow.

### 2.4. Neuroinflammation and Glial Cell State Reprogramming

The third axis—Neuroimmune-Glial Reprogramming—addresses how peripheral signals from the gut influence the brain’s resident immune cells. Neuroinflammation, especially from microglia, drives neurodegenerative diseases. In AD, microglia undergo morphological and functional changes in response to Aβ and tau pathology. Single-cell studies show that TREM2 drives protective microglial subgroups. But long-term activation wears them out, causing poor phagocytosis and more inflammation. The balance between protective and harmful microglial states controls how chronic neuroinflammation progresses in AD [17,18].

In addition, astrocytes also undergo phenotypic transformation in AD pathology and may exhibit either the neurotoxic A1 phenotype or the neuroprotective A2 phenotype [19]. The A1 phenotype is induced by interleukin-1α, tumor necrosis factor, and complement component 1q secreted by activated microglia [20]. This “glial reprogramming” idea gives a cell-level reason for how diets like the Mediterranean diet protect the brain. Lab and animal studies show that polyphenols can help neural stem cells grow and increase brain-derived neurotrophic factor (BDNF). This improves neuroplasticity. Olive polyphenols may also lower neuroinflammation by changing the silent information regulator 1/AMP-activated protein kinase and PI3K/Akt/mTOR signaling pathways. They also bind to metal ions and reduce oxidative damage from iron buildup in the brain [21].

Docosahexaenoic acid (DHA) blocks amyloid processing by lowering BACE1 and presenilin 1. It also stops fibril buildup by binding to the Aβ16-21 fragment. And it cuts tau phosphorylation through the JNK/GSK-3β pathway. DHA’s metabolites help clear Aβ and lower inflammation. Clinical studies suggest the benefits are bigger in APOE ε4 carriers and in people taking 500 mg/day or more of DHA for a long time [22]. Cross-disease relevance of glial reprogramming: While microglial TREM2-driven protective states are observed in both AD and PD models, as TREM2 expression is upregulated in these diseases serving a protective role through modulation of inflammatory signaling pathways [1], the specific triggers and temporal dynamics differ. In AD, amyloid plaques are the primary instigators of microglial activation, whereas in PD, α-synuclein aggregates and mitochondrial dysfunction in dopaminergic neurons appear to drive glial responses. This axis thus provides a cell-level explanation for how the Mediterranean diet might exert broad neuroprotective relationship, while also explaining disease-specific nuances [23]. Both TREM2-driven microglial state transitions and NF-κB/NLRP3 inflammasome modulation are supported by preclinical evidence—the former by human single-cell transcriptomic data and animal models, the latter by animal studies (both Tier II). However, direct evidence for Mediterranean diet-mediated modulation of these glial states in humans is currently unavailable and awaits validation in well-designed intervention trials.

### 2.5. Mitochondrial Function, Oxidative Stress, and Protein Homeostasis

Olive oil polyphenols represent one of the most extensively studied bioactive components of the Mediterranean diet, with multiple neuroprotective mechanisms as detailed below. The fourth axis—Proteostasis-Mitochondrial Quality Control—encompasses the direct cellular maintenance mechanisms that are modulated by Mediterranean diet components. Olive oil polyphenols like hydroxytyrosol (HT) and oleuropein may protect the brain indirectly by improving metabolic syndrome. These compounds help control glucose and lipid metabolism, reduce insulin resistance and chronic low-grade inflammation. This lowers oxidative stress and neuroinflammation in the brain, which helps keep neurons stable [24]. Collectively, these actions contribute to the suppression of neuroinflammation and the delay of neurodegenerative progression.

Computational biology studies further suggest that polyphenols in EVOO can activate the Nuclear factor erythroid 2-related factor 2 (NRF2) pathway, thereby reducing BACE1 and Aβ deposition, whereas β-sitosterol may stabilize mitochondrial function through binding to Aβ and downregulating GSK-3β, among other targets. Population-based studies have also confirmed the benefits of β-carotene and lycopene for brain health. Taken together, oleuropein, HT, and oleocanthal (OC) appear to act through complementary mechanisms to preserve proteostasis and mitochondrial function [18].

Molecular simulation has further confirmed that oleuropein aglycone (OA) can disaggregate Aβ fibrils, HT can promote the formation of non-toxic Aβ conformations, and OC can facilitate Aβ clearance while also inhibiting tau fibrillization [25]. In addition, polyphenols such as oleuropein can upregulate hepatic expression of low-density lipoprotein receptor-related protein 1, a key mediator of circulating Aβ uptake and clearance, suggesting that polyphenols in the Mediterranean diet may indirectly reduce cerebral Aβ deposition by enhancing peripheral hepatic clearance of Aβ [26].

Computational biology studies have identified the NRF2 pathway as a main target of EVOO polyphenols. Apigenin and luteolin may activate NRF2. This reduces BACE1, β-CTF, and Aβ deposition, and improves cognitive function. Luteolin may also lower Aβ production through PPAR-γ. Caffeic acid may reduce amyloid precursor protein (APP) and BACE1 expression, which lowers Aβ1-42 levels. These compounds are structurally similar to known NRF2 activators (Tanimoto coefficient > 0.9). They are also linked to pathways involved in neuroimmune changes. This suggests that NRF2 is a key target through which these bioactive compounds protect the brain at a systems level [27].

Molecular docking studies show that β-sitosterol binds strongly to Aβ aggregates. In AD animal models, giving wheat germ oil rich in β-sitosterol lowered GSK-3β and APP expression. It also raised Akt, reduced TNF-α (an inflammatory cytokine linked to neuroimmune changes) and malondialdehyde (a marker of oxidative stress), and helped stabilize mitochondrial–metabolic coupling [28].

A cross-sectional neuroimaging study looked at 132 people aged 30 to 50 years. It found that higher plasma β-carotene levels were linked to a “younger” brain age measured by MRI. The standardized regression coefficient was −0.23. For each 1-log-unit increase in β-carotene, brain age was 1.46 years younger. This suggests that β-carotene may protect the brain through a healthy gut microbiota. It may also help stabilize mitochondrial–metabolic coupling and slow down biological brain aging [29]. A Japanese longitudinal study followed 199 people aged 39 to 90 years for 5 years. It found that low serum lycopene levels were linked to faster decline in attention. The slope coefficient was −3.17 (*p* = 0.002) on the Digit Cancellation Test, version 3. β-carotene showed no clear link to attention decline. These findings suggest that lycopene may protect attention by changing microglial states and reducing abnormal neuroimmune changes [30].

Phospholipids and omega-3 phospholipids are good long-term sources of n-3 PUFAs in the Mediterranean diet. Cooked mussels (*Mytilus edulis*) contain about 518.9 ± 155.7 mg/100 g of EPA + DHA. Eating mussels three times a week for two weeks raises the Omega-3 Index and blood EPA levels in people. The n-3 PUFAs from mussels have direct anti-inflammatory and antioxidant effects. They also help the gut–immune–brain axis by changing the gut microbiota. In addition, farmed mussels produce much less carbon than meat or farmed fish. They do not harm fish stocks. This fits with the green and sustainable principles of the Mediterranean diet [31].

Current preclinical evidence shows that EVOO polyphenols work against AD through several targets. HT and oleuropein block Aβ and tau aggregation. OC helps clear Aβ across the BBB. Verbascoside and OC lower neuroinflammation. HT and related compounds help fix mitochondrial function and cut oxidative stress. Many polyphenols also improve neuroplasticity and synaptic function [32]. These mechanisms help keep mitochondrial–metabolic coupling working, lower oxidative stress, and control proteostasis. HT mainly changes oxidative stress, neuroinflammation, and mitochondrial bioenergetics. OC has the strongest anti-Aβ and anti-tau effects, including helping clear Aβ across the BBB [33].

Beyond the canonical NF-κB/NLRP3 inflammasome pathways, the cGAS-STING/type-I interferon axis has emerged as a central integrator of neuronal stress and neuroimmune dysfunction in neurodegenerative diseases. Mitochondrial DNA released from stressed neurons activates cGAS, leading to STING-dependent type-I interferon responses that promote synaptic loss and cognitive decline. Given that Mediterranean diet polyphenols protect mitochondrial integrity and reduce oxidative stress, it is plausible that these compounds indirectly suppress cGAS-STING activation by mitigating mitochondrial damage—a hypothesis that warrants direct experimental testing in future dietary intervention studies [34]. The mechanistic evidence for EVOO polyphenols described above falls into distinct evidence tiers. The anti-Aβ and anti-tau activities are supported primarily by in vitro and molecular simulation studies, with limited supportive data from human studies (Tier II–III); larger confirmatory studies are required. By contrast, NRF2 pathway activation is primarily derived from computational docking and in vitro studies (Tier III), and the cGAS-STING pathway as a mediator of diet-induced neuroprotection remains an emerging hypothesis based on preclinical evidence (Tier III). Collectively, these findings highlight that while olive polyphenols show promise through multiple mechanisms, the majority of these pathways require in vivo validation and larger human confirmatory studies.

### 2.6. BBB and Neurovascular Unit

The Trp–AhR pathway is a major signaling axis in the gut–immune–brain network. AhR levels are higher in the hippocampus of AD patients. Gut bacteria imbalance can produce uremic toxins like indoxyl sulfate. These toxins activate AhR, disrupt the BBB, and cause cognitive problems. The Mediterranean diet is rich in tryptophan and fiber. It may improve microbial metabolism and cut down harmful AhR agonists. Its polyphenols, like resveratrol, also block AhR activity. So changing the Trp–AhR pathway may be a new way the Mediterranean diet protects the brain through the gut–brain axis [35]. High adherence to the Mediterranean diet is associated with neuroprotective relationship [7].

The Mediterranean diet is thought to act through several pathways at once. The diet also helps reshape the gut microbial ecosystem. Through the gut–immune–brain axis, it fixes dysregulated neuroimmune changes and protects the BBB. It may also improve metabolic health and lower vascular risk factors. This indirectly cuts the risk of neurodegenerative diseases [36].

**Figure 2:**
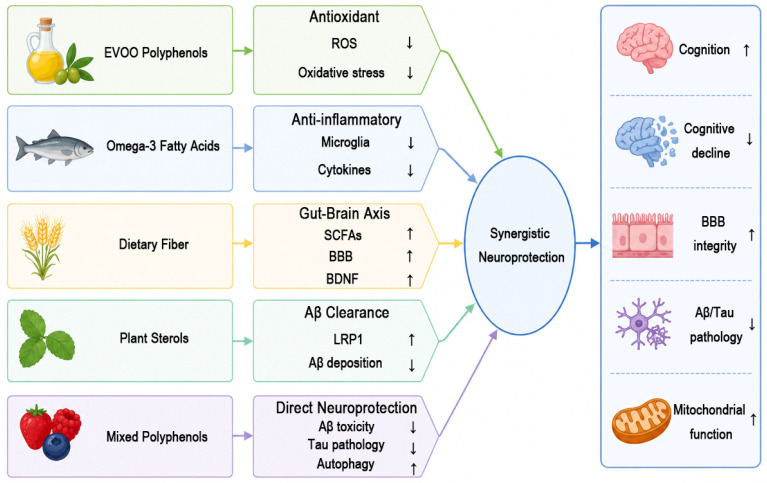
Synergistic neuroprotective mechanisms of Mediterranean diet-derived bioactive components in neurodegenerative disorders. Schematic overview illustrating how major Mediterranean diet-associated bioactive components cooperatively regulate multiple neuroprotective pathways involved in neurodegenerative disease prevention and progression. Distinct dietary factors, including EVOO-derived polyphenols, n-3 PUFAs, dietary fiber, plant sterols, and mixed polyphenolic compounds, modulate complementary biological pathways associated with oxidative stress reduction, neuroinflammation suppression, gut–brain axis regulation, Aβ clearance, and direct neuronal protection. These interconnected mechanisms converge to promote synergistic neuroprotection through attenuation of reactive oxygen species, suppression of microglial activation and pro-inflammatory cytokine production, enhancement of SCFA generation and BBB integrity, facilitation of Aβ clearance pathways, and improvement of autophagic and mitochondrial homeostasis. Collectively, these integrated effects contribute to preserved cognitive function, delayed cognitive decline, maintenance of BBB integrity, reduced Aβ/tau pathology, and improved mitochondrial function during neurodegenerative disease progression.

Beyond these core pathways, the Mediterranean diet may also lower BACE1 activity through the Wnt/β-catenin/TCIM pathway. This reduces Aβ production and tau hyperphosphorylation. The diet may also help clear Aβ by improving insulin resistance and boosting insulin-degrading enzyme efficiency. At the same time, it may change the expression of cognition-related genes in the brain, improve cognitive resilience, and slow down dementia progression [37]. These mechanisms do not operate independently; rather, they constitute a cascading network extending from the gut to the central nervous system, collectively mediating the multitarget neuroprotective relationship of the Mediterranean diet (Figure 2). The Trp-AhR-BBB pathway is supported by human AD hippocampal data and animal models (Tier II); the specific effect of Mediterranean diet on this pathway in humans requires further investigation.

## 3. Disease-Specific Research Advances Within a Shared Mechanistic Framework

### 3.1. AD and All-Cause Dementia

Studies show that sticking to the Mediterranean diet for a long time is linked to a lower risk of AD. The more people follow it, the lower the risk. Eating olive oil, fish, and vegetables is also linked to better cognitive function. The MIND diet was designed to prevent neurodegenerative diseases. It combines key features of the Mediterranean and DASH diets. People who follow the MIND diet closely also have lower risks of dementia and AD. Their cognition and memory also stay better. But the effects seem to vary across populations. The evidence is more consistent in North American groups [38]. A systematic review of five studies found that high adherence to the MIND diet was linked to less cognitive impairment. It also came with better brain biomarkers and cognitive scores. This suggests the MIND diet may improve cognitive function in older adults [39]. Data from the UK Biobank cohort show that high adherence to the Mediterranean diet is linked to a lower risk of all-cause dementia. The effect is stronger in older people, women, and non-carriers of APOE ε4. In contrast, pro-inflammatory diets are linked to a higher risk of dementia. This highlights the value of high-quality anti-inflammatory diets for brain health [40].

More detailed analyses from the same UK Biobank cohort confirmed that high adherence to the Mediterranean diet was linked to a 23% lower risk of all-cause dementia (hazard ratio = 0.77). This protective relationship was independent of polygenic risk, including APOE ε4 genotype. A continuous adherence score for the Mediterranean diet also seemed to work better than the traditional binary scoring method [41]. However, pooled evidence from multiple large prospective cohorts indicates that the protective relationship of the Mediterranean diet on dementia risk shows a degree of heterogeneity, which may be attributable to differences in study populations, duration of follow-up, and methods of dietary assessment [42]. The Nordic diet, another healthy pattern, may also delay cognitive decline and dementia through similar anti-inflammatory and neuroprotective mechanisms as the Mediterranean diet, though evidence is still limited [43].

### 3.2. Parkinson’s Disease

Studies on the Mediterranean diet and PD are still limited. But the available evidence suggests a protective relationship. A 2025 meta-analysis included seven observational studies with 195,065 people and 1508 PD cases. It found that high adherence to the Mediterranean diet was linked to a 13% lower risk of PD. The pooled relative risk was 0.87 with a 95% confidence interval of 0.78 to 0.97 [44]. High adherence to a healthy plant-based diet, with lots of vegetables, nuts, and tea, was linked to a 22% lower risk of PD. This protective relationship was changed by the polygenic risk score for PD. In contrast, an unhealthy plant-based diet had the opposite effect [45].

At the mechanistic level, PD patients have clear changes in their gut microbiota. SCFA-producing bacteria like *Faecalibacterium*, *Roseburia*, and *Lachnospiraceae* are much lower. This leads to less butyrate and other SCFAs, which hurts the gut barrier and worsens inflammation in the body and brain. In contrast, pro-inflammatory bacteria like *Enterococcaceae* and *Christensenellaceae* are higher. Their endotoxins may damage the gut lining. Less *Prevotella* is also linked to more severe PD, so it may be a biomarker. These gut changes help explain how the Mediterranean diet could affect PD by reshaping the gut microbiota [46].

A systematic review looked at studies published up to June 2023. It included one randomized controlled trial with 70 people, one case–control study with 8 people, and one cohort study with 1205 people. The review found that high adherence to the Mediterranean diet improved non-motor symptoms in PD patients. These included executive function, language, attention, working memory, overall cognition, and gut issues like constipation and dyspepsia. But motor symptoms like tremor, bradykinesia, and postural instability did not improve [47]. A neuroimaging meta-analysis including 13 cross-sectional studies and a total of 42,955 participants found that high adherence to the Mediterranean diet was significantly associated with lower white matter hyperintensity volume, but showed no clear association with total brain volume, gray matter volume, or hippocampal volume. These findings suggest that the Mediterranean diet may reduce dementia risk, at least in part, by protecting the cerebral small vessels [48].

### 3.3. Other Neurodegenerative Diseases

Beyond AD and PD, the potential protective relationship of the Mediterranean diet on other neurodegenerative disorders, including amyotrophic lateral sclerosis (ALS) and multiple sclerosis (MS), are also beginning to be explored.

With respect to ALS, a 12-month prospective study showed that a Mediterranean diet intervention changed SCFA levels in the blood, including acetate and propionate. The study also found that these changes in SCFAs were linked to ALS disease progression over time. But whether the diet directly affects clinical outcomes still needs to be tested in larger studies [49].

Regarding MS, a mechanistic review suggested that the Mediterranean diet may affect the inflammatory and neurodegenerative processes of MS by changing the network of immunometabolites like citrate, itaconate, and glutamate. These metabolites are involved in immune cell changes [50]. Animal studies have provided more causal evidence. In a mouse model of experimental autoimmune encephalomyelitis, a combined intervention with the Mediterranean diet and lycopene slightly delayed disease onset and clearly lowered clinical scores. Myelin staining showed that the myelination score in the combined intervention group (MD-Lyc) was higher than in both the Western diet group (WD) and the Western diet plus lycopene group (WD-Lyc). Mechanistic analyses also showed that this diet changed gut microbiota composition. Mice in the MD-Lyc group had more beneficial bacteria like *Akkermansia* and *Bifidobacterium*, and fewer potentially harmful bacteria like *Helicobacter* and Pseudomonas. These findings suggest that the gut–brain axis may mediate how High adherence to the Mediterranean diet is associated with neuroprotective relationship in MS [51].

Taken together, the clinical evidence for the Mediterranean diet in ALS and MS is still less strong than for AD and PD. But existing mechanistic studies and animal experiments have shown promising relationship. These early findings suggest that the neuroprotective relationship of the Mediterranean diet may apply broadly across neurodegenerative diseases. The core mechanisms are likely tied to the gut microbiota–immune–metabolic network (Table 1).

### 3.4. Cross-Disease Mechanistic Comparison: Shared Pathways and Disease-Specific Nuances

While the four mechanistic axes described above are broadly relevant across neurodegenerative diseases, the relative contribution of each axis varies by disease context. In AD, Aβ and tau pathology are strongly linked to the Proteostasis-Mitochondrial Axis (Axis 4), with extensive evidence for microglial involvement (Axis 3). In PD, the gut-first versus brain-first hypothesis places particular emphasis on the Microbiome-Metabolite Axis (Axis 1) and Gut Barrier-Systemic Inflammation Axis (Axis 2), given the prominent gastrointestinal prodromal symptoms [52]. In MS, the autoimmune nature of the disease highlights the Neuroimmune-Glial Axis (Axis 3), while ALS research has increasingly focused on mitochondrial dysfunction and proteostasis (Axis 4) [53]. Understanding these disease-specific emphases is crucial for designing targeted dietary interventions and interpreting the heterogeneous epidemiological evidence summarized in Table 1.

Beyond neurodegenerative diseases, the Mediterranean diet has been associated with reduced risk of cardiovascular disease, colorectal cancer, type 2 diabetes, and metabolic dysfunction-associated steatotic liver disease [54]. These conditions share chronic inflammation and metabolic dysregulation as common pathophysiological features, reinforcing the concept that neurodegenerative and metabolic diseases share convergent biological pathways. This cross-disease perspective supports the view that the Mediterranean diet operates through fundamental systems-level mechanisms—namely, inflammation control, oxidative stress reduction, and metabolic homeostasis—rather than through highly disease-specific pathways.

**Table 1:** Summary of epidemiological evidence regarding the Mediterranean diet across different neurodegenerative diseases.

Disease	Study Design	Sample Size	Dietary Assessment	Main Outcome	Effect Size (95% CI or HR)	Limitations	Evidence Level
AD [36]	Meta-analysis of cohort studies	Variable	MDS, aMED	Lower risk of AD with high adherence	RR 0.64 (0.46–0.89)	High heterogeneity; residual confounding	Tier I
All-cause dementia [41]	Prospective cohort (UK Biobank)	*n* = 131,209	aMED	23% lower risk with high adherence	HR 0.77 (0.66–0.90)	Self-reported diet; predominantly European ancestry	Tier I
PD [44]	Meta-analysis of 7 observational studies	*n* = 195,065	Various	13% lower risk with high adherence	RR 0.87 (0.78–0.97)	Only 7 studies; limited RCT data	Tier I
Cognitive decline [1]	Prospective cohort (PREDIMED)	*n* = 447	MDS	Slower cognitive decline in high adherence group	β = 0.11 (*p* < 0.05) per MDS point	Specific to Mediterranean population; short follow-up	Tier I
MCI to AD progression [55]	RCT (EVOO intervention)	*n* = 70	Intervention (30 mL/day EVOO)	Improved cognitive function; reduced Aβ42/Aβ40 ratio	*p* < 0.05 for both outcomes	Short duration (6 months); small sample size	Tier II
ALS [49]	Prospective observational	*n* = 12	Intervention (MedDiet)	Changes in SCFA profiles linked to progression	Correlation analysis only	Very small sample; no clinical outcome data	Tier III

## 4. Emerging Technologies Reshaping the Field

### 4.1. Multi-Omics Technologies: From Single-Cell to Spatially Resolved In Situ Analysis

Multi-omics combines high-throughput data from the genome, transcriptome, proteome, metabolome, microbiome, and other layers. In nutrition and neuroscience, its main value is moving Mediterranean diet research from asking “does it work” to explaining “how it works” and “where it works” at the cell and tissue level. In short, it shifts the focus from broad diet patterns to specific molecular pathways, cell types, and spatial settings. Below are the main applications of single-cell transcriptomics, spatial transcriptomics, and microbiome multi-omics in this field.

#### 4.1.1. Single-Cell Transcriptomics: Precise Analysis of Cell Subpopulations

Single-cell transcriptomics is a high-throughput method that measures the complete transcriptome of individual cells. Unlike bulk RNA-seq, which averages gene expression across many cells, this method shows the gene expression profile of each cell. This helps identify different cell subtypes and functional states within mixed cell populations. In neurodegenerative disease research, single-cell transcriptomics can reveal how the Mediterranean diet affects specific cell types. Its main value is picking out key responsive cell groups from averaged signals. In short, this approach allows subtype-level analysis and directly identifies which glial cell types and state changes are regulated by the Mediterranean diet [56].

For instance, single-cell transcriptomic studies have identified TREM2-driven protective microglial subpopulations in AD models, providing cellular-level resolution to the anti-inflammatory mechanisms discussed in Section 2.4. This technology allows researchers to determine whether dietary interventions modulate the balance between protective and harmful microglial states—a question that cannot be addressed by bulk tissue analysis [14,15].

#### 4.1.2. Spatial Transcriptomics: From Spatial Localization to Functional Dissection

Spatial transcriptomics measures gene expression while keeping the spatial location of cells in intact tissue. Conventional RNA sequencing requires breaking down tissue, which loses spatial information. In contrast, spatial transcriptomics can directly show where specific genes are expressed on tissue sections and how much they are expressed. In neurodegenerative disease research, this method can identify inflammatory spots around Aβ plaques in place. It can show that damage is concentrated in the upper cortical layers. It can also test whether dietary interventions improve the disease environment by measuring changes in inflammatory gene expression near plaques. So it provides spatial evidence that dietary interventions protect the brain [57].

Spatial transcriptomics has enabled in situ mapping of inflammatory gene expression around Aβ plaques in AD brains, identifying zone-specific transcriptional signatures involving microglial and astrocytic responses. The SpaHDmap framework further upgrades spot-level expression data to pixel-level resolution by integrating spatial transcriptomic gene expression with high-resolution histology images, allowing precise localization of gene expression to hippocampal subregions (CA1-CA3, dentate gyrus) and cortical layers (L1-L6b). Such spatial resolution offers unprecedented opportunities to test whether dietary interventions alter the inflammatory microenvironment in specific brain regions [55].

For example, spatial transcriptomics has enabled in situ mapping of inflammatory gene expression around Aβ plaques in human AD brains, revealing spatially organized glial responses including disease-associated microglia (DAM) and reactive astrocyte signatures. This spatial resolution offers a direct approach to test whether Mediterranean diet components alter the inflammatory microenvironment in plaque-associated regions, as discussed in Section 2.4 and Section 2.5 [57].

Spatial transcriptomic analyses have revealed a ‘core-shell’ cellular organization pattern around Aβ plaques: microglia aggregate near the plaque core, while astrocytes are distributed more peripherally, and proximal regions are enriched for DAM markers including Cst7, Itgax, and Apoe, whereas distal regions retain a more homeostatic state [58].

#### 4.1.3. Microbiome Multi-Omics: Multi-Layer In-Depth Interrogation of Gut Microbiota

Microbiome multi-omics combines several high-throughput methods like metagenomics, metatranscriptomics, metaproteomics, and metabolomics. It looks at the composition, function, and metabolic products of microbial communities. Traditional 16S rRNA sequencing mostly tells you “who is there.” Microbiome multi-omics goes further to ask “The functional activities of the microbiota” This method can directly find key functional metabolites like SCFAs that come from diet-driven gut microbial activity. It can also combine multi-omics data to link these metabolites to health benefits for the host [59].

For example, the METRIC (Microbiome-based nutrient profile corrector) model uses gut metagenomic profiles to correct random errors in self-reported dietary data derived from 24 h recalls or diet records. Using a skip-connection deep learning architecture, the model adds the corrupted nutrient profile directly to the final network output, preventing overcorrection. Simulation and validation studies demonstrated excellent correction performance, particularly for nutrients metabolized by gut bacteria, in both synthetic and three real-world datasets [57]. This demonstrates how microbiome multi-omics can complement self-reported dietary assessment and reduce measurement error in dietary intake, enhancing the reliability of nutritional epidemiology in large-scale cohorts [60].

More directly relevant to the gut–brain axis mechanisms elaborated in Section 2.1, Section 2.2 and Section 2.3, combined microbiome and dietary profiling has proven critical for establishing a link between adherence to the Mediterranean diet and better cognitive performance. For example, a six-year prospective cohort study leveraging 16S rRNA sequencing found that greater compliance with the Mediterranean diet correlated with elevated levels of short-chain fatty acid (SCFA)-generating bacteria such as *Barnesiella*, *Butyricicoccus*, and *Agathobacter*. This research also constructed a microbiota-derived Mediterranean diet adherence score (MedDiet-GMS), which was independently predictive of attenuated overall cognitive deterioration and sustained executive function across the six-year follow-up period [7]. Though the observational nature of this work cannot confirm causal relationships, this multi-omics analysis delivers correlational evidence that dietary habits remodel gut microbial composition to yield beneficial cognitive outcomes, aligning with the gut–brain axis paradigm outlined throughout this review.

#### 4.1.4. Validation and Application of Multi-Omics Integration Strategies

Multi-omics integration jointly analyzes data from genomics, transcriptomics, proteomics, metabolomics, and microbiomics. It looks at how different molecular layers work together instead of looking at one dataset alone. In nutrition and neuroscience, its main value is showing how dietary interventions affect the brain through multiple targets and pathways at once.

The value of this multi-omics approach is shown by the genetic resources from the MIND trial. A genetic substudy did genome-wide genotyping on MIND trial participants and improved quality control. After strict quality control, high-quality genetic data were kept for 494 people of European ancestry and 58 of African ancestry. Importantly, the study compared DNA from serum and whole blood from the same people and found a match rate over 99.2%. This confirms that serum is a reliable DNA source when whole blood is not available. These resources provide a strong base for building polygenic risk scores, studying gene–diet interactions, and doing multi-omics integration [61].

Proteomic studies have revealed that many AD-associated molecular alterations are more pronounced, or even uniquely detectable, at the protein level, and deep multi-layer analyses demonstrate that protein-level changes often diverge significantly from transcriptional patterns, reflecting post-transcriptional regulation, protein turnover, and pathway activity states.

Data from the same MIND trial challenge the simple idea of grouping people just by APOE ε4 status. A genome-wide analysis of 604 participants found that APOE ε4 itself did not change how much the MIND diet helped cognitive function. This suggests that using only APOE ε4 to group people may be too simple. So the study called for more complete gene–diet interaction analyses using genome-wide data. It also pushed for a move from “single-gene stratification” to “genome-wide polygenic risk score-based stratification” [62].

A practical example of multi-omics integration in precision nutrition comes from the MIND trial genetic resource. This substudy performed genome-wide genotyping on 604 participants using DNA from whole blood or serum, and demonstrated that genotype concordance between serum and blood DNA exceeded 99.2%, validating serum as a reliable alternative when whole blood is unavailable. The study further revealed that APOE ε4 alone did not significantly modify cognitive responses to the MIND diet, suggesting that single-gene stratification is insufficient for capturing individual variability in dietary response. Instead, genome-wide polygenic risk scores provide a more comprehensive framework for gene-diet interaction analyses. This genetic resource, available through NIAGADS, enables integrative analyses with other omics data—including transcriptomics, metabolomics, and microbiome—to elucidate mechanisms underlying cognitive decline and dietary response [55]. Collectively, these findings demonstrate how genomic approaches can move beyond population averages to identify subtype-specific responses, advancing precision nutrition for cognitive health [61].

In summary, multi-omics operationalizes the four mechanistic axes: microbiome multi-omics informs Axis 1 (microbial composition and functional outputs); metabolomics/proteomics inform Axis 2 (inflammation and barrier integrity); single-cell transcriptomics resolves Axis 3 (protective vs. harmful glial states); spatial transcriptomics with proteomics/docking operationalizes Axis 4 (protein aggregates and mitochondrial stress); genomics provides host genetic context (APOE ε4, PRS) for subtype stratification. Collectively, these tools translate theoretical axes into testable underscore frameworks, advancing personalized precision nutrition for neurodegenerative disease prevention.

### 4.2. Precision Nutrition and Artificial Intelligence (AI): From Population Stratification to Personalized Intervention

In recent years, precision nutrition and nutritional neuroscience have come together to form a new research framework. The goal is to design personalized diet plans based on a person’s multi-omic profile and daily habits. This is meant to improve neural resilience. Neural resilience is the brain’s ability to keep working and stay flexible under stress, injury, or disease [63]. This research approach uses multi-omics technology. It moves beyond just testing whether the Mediterranean diet works in large groups. Some studies have looked at how the diet works and who it helps at the individual level. By combining genomics, microbiomics, and metabolomics, researchers can classify people more precisely and target interventions. This gives strong theoretical and technical support for developing and using precision nutritional neuroscience in the clinic [59].

#### 4.2.1. Multi-Omics-Based Individual Stratification Strategies

The neuroprotective relationship of polyphenols like ellagic acid and ellagitannins depend on gut bacteria breaking them down into urolithins. People can be grouped by this ability into urolithin A producers, urolithin B producers, or non-producers. Urolithin A producers have a healthier gut profile. Urolithin B producers are more common in older people and have more dysbiosis. Urolithin A improves muscle strength, lowers neuroinflammation, and boosts cognition. The same idea applies to soy isoflavones. Only people with the right gut bacteria can turn them into equol and get the brain benefits. So differences in how people respond to polyphenols in the Mediterranean diet come down to their gut bacteria. This gives a new way to group people for precision nutrition [64].

#### 4.2.2. Computational Nutrition and AI-Assisted Technologies

AI and computational nutrition combine multi-omics data like genomics, microbiomics, and metabolomics. They build models to predict how different people will respond to the Mediterranean diet. Then researchers can group people based on APOE ε4 genotype, gut microbial enterotype, and baseline metabolic status. This allows personalized interventions. These tools provide key support for precision nutrition strategies in neurodegenerative diseases [65]. This integrated analytical framework has also been methodologically validated in functional food research.

One study used network pharmacology to find 45 AD-related targets of *Pueraria lobata* (kudzu root). These targets were linked to synaptic plasticity pathways. Mendelian randomization then confirmed a clear link between PFKFB3 and AD. Molecular docking showed that the active compounds bind strongly to their targets. This shows a complete “food–target–disease” research pipeline [66]. AI can pull together different types of data in computational nutrition to find high-risk phenotypes. Image recognition allows real-time tracking of food intake. Wearable devices help collect digital phenotypes. Machine learning can analyze neuroimaging biomarkers. AI also has potential uses in food classification and cognitive modeling. But challenges remain. These include algorithmic bias, data privacy, and model interpretability. Addressing these issues is needed to make sure the tools work fairly across different populations [67].

## 5. Limitations and Future Directions

### 5.1. Current Status and Future Perspectives of Randomized Controlled Trials (RCTs)

Most current research is observational. Large, long-term randomized controlled trials (RCTs) are still rare. So we lack strong causal evidence on how the Mediterranean diet affects the progression of neurodegenerative diseases. The way different bioactive components like polyphenols, n-3 PUFAs, and fiber work together is not fully understood. Their best intake levels, ratios, and interactions also need more study in large prospective trials [68]. Notably, a 6-month RCT has provided preliminary evidence that daily consumption of 30 mL of EVOO can significantly improve cognitive function in patients with mild cognitive impairment while also reducing the plasma Aβ42/Aβ40 ratio and the p-tau/tau ratio and improving BBB permeability as well as brain functional connectivity [55]. These findings provide both a rationale and a basis of feasibility for conducting larger-scale, long-term RCTs.

### 5.2. Genetic Polymorphisms and Individual Heterogeneity

Large-scale prospective cohort studies have confirmed that carriers of the APOE ε4 allele differ in their neuroprotective response to the Mediterranean diet, suggesting that future dietary strategies should be tailored according to genotype [69]. Beyond APOE ε4, the COMT Val158Met polymorphism significantly interacts with the MIND diet. Met/Met carriers have about 40% lower enzyme activity and showed greater perceptual speed improvement on the diet, whereas no clear effects were seen in Val carriers. COMT metabolizes dopamine and polyphenols such as HT and anthocyanins. Lower enzyme activity in Met/Met carriers may reduce polyphenol breakdown and enhance their bioavailability. These findings suggest that COMT genotyping could inform precision nutrition, with Met/Met carriers benefiting most from higher polyphenol intake [62].

### 5.3. Complementary Mechanisms of Functional Components: A Case Study of Olive Oil Polyphenols

The various functional components of the Mediterranean diet exhibit intrinsic mechanistic complementarity. For example, oleuropein/OA, HT, and OC target distinct neuroprotective pathways. Oleuropein primarily acts on protein aggregation, mitochondrial protection, and autophagy. HT mainly regulates oxidative stress, neuroinflammation, and mitochondrial bioenergetics. OC, in contrast, demonstrates the strongest anti-Aβ and anti-tau activities, including promotion of Aβ clearance across the BBB [70]. In addition, neuroimaging studies have shown that diet may exert complementary structural effects by modulating white matter connectivity, hippocampal volume, and cortical thickness, although the underlying mechanisms remain to be further elucidated [71]. Therefore, in-depth investigation of the complementary mechanisms among different dietary components may help enhance neuroprotective efficacy at the level of precision nutrition in the future.

### 5.4. Precision Nutrition Strategies: From Multi-Omics to Chrononutrition

Precision nutrition is a new field. It designs personalized diet plans based on differences in genes, gut microbiota, metabolism, lifestyle, and environment. It brings together nutrigenomics, metabolomics, and microbiomics to tailor diets to a person’s genotype and microbial profile. Chrononutrition strategies like time-restricted eating can change the gut microbiota, boost beneficial bacteria such as *Bifidobacterium pseudolongum*, and increase propionate production. This activates free fatty acid receptor 3 and improves cognitive function. These findings show that meal timing plays a key role in protecting the brain [65]. Clinical translation of precision nutrition faces practical and ethical barriers, including high multi-omics costs that exacerbate disparities, a lack of validated biomarkers for patient stratification, complex data interpretation, and limited clinician training. Genetic risk communication risks psychological harm and discrimination. These barriers must be addressed alongside scientific advances for equitable implementation [72].

### 5.5. The “Double-Edged Sword” Effect: Caveats Regarding “One-Size-Fits-All” Intervention Strategies

Precision nutrition needs to watch out for the risks of a “one-size-fits-all” approach. The same gut bacteria or metabolites can have opposite effects in different diseases. For example, SCFAs from gut bacteria may promote Aβ deposition in germ-free conditions. TMAO from microbial breakdown of red meat can make AD worse. But IPA, a tryptophan-derived microbial metabolite, protects the brain. Also, *Akkermansia* seems to protect against AD but is linked to higher risk in PD. These “double-edged sword” effects show that future research should set different microbial or metabolite targets based on the specific disease, genetic background, and baseline health status. A universal strategy may not be optimal for all individuals [73].

### 5.6. International Expert Consensus and Future Research Priorities

**Figure 3:**
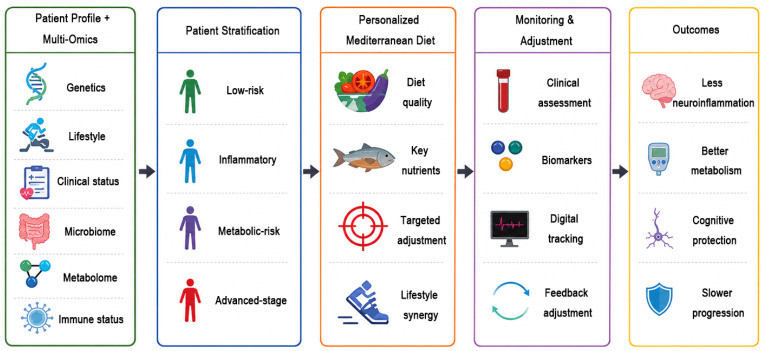
Precision nutrition framework integrating multi-omics profiling and personalized Mediterranean diet intervention for neurodegenerative disease management. Schematic overview illustrating a precision nutrition strategy for individualized neuroprotective intervention based on integrated multi-omics profiling. Comprehensive patient characterization incorporates genetic background, lifestyle factors, clinical phenotypes, gut microbiome composition, metabolomic signatures, and immune status to establish multidimensional disease-associated profiles. Patients are subsequently stratified into distinct pathological or risk-associated subgroups, including low-risk, inflammatory-dominant, metabolic-risk, and advanced-stage phenotypes. Based on these classifications, personalized Mediterranean diet intervention strategies are designed through optimization of dietary quality, targeted nutrient supplementation, precision dietary adjustment, and synergistic lifestyle interventions. Longitudinal monitoring is achieved through clinical assessment, biomarker evaluation, digital health tracking, and adaptive feedback-based optimization to dynamically refine intervention efficacy. Collectively, this integrated framework aims to reduce neuroinflammation, improve metabolic homeostasis, preserve cognitive function, and delay neurodegenerative disease progression through individualized dietary modulation and systems-level precision medicine approaches.

An international consensus outlined nutrition–brain health priorities: standardized cognitive measures, long-term early-intervention trials, multidomain FINGER-type strategies, vulnerable population inclusion, AI-driven dietary tools, and patient/caregiver-informed design. These priorities collectively shift the field from short-term, single-component to long-term, multidomain precision approaches [72] (Figure 3).

### 5.7. Methodological Limitations in Dietary Assessment

Most prospective studies rely on self-reported dietary intake via food frequency questionnaires, which are subject to measurement errors and recall bias that may attenuate true relationship between Mediterranean diet adherence and cognitive outcomes [68].

## 6. Conclusions

Current epidemiological and mechanistic studies consistently indicate that the Mediterranean diet exerts a clear neuroprotective relationship against neurodegenerative diseases such as AD and PD through anti-inflammatory, antioxidant, gut–brain axis modulation, and multitarget synergistic mechanisms. Among its components, olive oil polyphenols, n-3 PUFAs, and dietary fiber appear to be the most critical bioactive constituents. Their effects involve molecular mechanisms such as inhibition of the NF-κB/NLRP3 inflammatory pathway, activation of the NRF2 antioxidant system, promotion of SCFA production, and regulation of the tryptophan–AhR axis.

However, most current evidence comes from observational studies, while large-scale, long-term RCTs are still lacking. Protective relationships are significantly influenced by factors such as genetic background (including APOE ε4 status), gut microbiota characteristics, and food processing methods, which limit both the standardization and personalization of intervention strategies. Future research should integrate nutrigenomics, metabolomics, and microbiomics to advance precision nutrition strategies and the development of functional foods, while also paying close attention to the impact of cooking and processing on bioactive compounds. Such efforts will help move the Mediterranean diet from a broad “dietary pattern” toward an evidence-based neuroprotective intervention.

## Data Availability

No new data were created or analyzed in this study. Data sharing is not applicable to this article.

## Abbreviations

AD	Alzheimer’s disease
AhR	Aryl hydrocarbon receptor
Akt	Protein kinase B
ALS	Amyotrophic lateral sclerosis
APOE ε4	Apolipoprotein E ε4 allele
APP	Amyloid precursor protein
Aβ	Amyloid-beta
BACE1	β-secretase 1
BBB	Blood–brain barrier
COMT	Catechol-O-methyltransferase
DASH	Dietary Approaches to Stop Hypertension
DHA	Docosahexaenoic acid
EPA	Eicosapentaenoic acid
EVOO	Extra virgin olive oil
GSK-3β	Glycogen synthase kinase-3β
HT	Hydroxytyrosol
IL-1β	Interleukin-1β
IL-6	Interleukin-6
IPA	Indole-3-propionic acid
MIND	Mediterranean-DASH Intervention for Neurodegenerative Delay
MS	Multiple sclerosis
NF-κB	Nuclear factor-κB
NLRP3	NOD-like receptor family pyrin domain-containing protein 3
NRF2	Nuclear factor erythroid 2-related factor 2
OA	Oleuropein aglycone
OC	Oleocanthal
PD	Parkinson’s disease
SCFAs	Short-chain fatty acids
TMAO	Trimethylamine N-oxide
TNF-α	Tumor necrosis factor-α
TREM2	Triggering receptor expressed on myeloid cells 2
Trp–AhR	Trp–AhR Tryptophan–Aryl Hydrocarbon Receptor
RCTs	Randomized Controlled Trials
n-3 PUFAs	omega-3 fatty acids
